# Recruitment of bone marrow CD11b^+^Gr-1^+^ cells by polymeric nanoparticles for antigen cross-presentation

**DOI:** 10.1038/srep44691

**Published:** 2017-03-20

**Authors:** Ya-Wun Yang, Wen-Hui Luo

**Affiliations:** 1 School of Pharmacy, College of Medicine, National Taiwan University, 33 Linsen South Road, Room 423, Taipei City 10050, Taiwan

## Abstract

The objective of this study was to investigate the function of poly(lactic-co-glycolic acid) (PLGA) nanoparticles (NPs) on the activation of antigen-specific CD8^+^ T cell responses via the CD11b^+^Gr^−^1^+^ myeloid subpopulations in murine bone marrow (BM). PLGA NPs containing ovalbumin (OVA) were fabricated by the double-emulsion method. The CD11b^+^Gr-1^low^Ly-6C^high^ and CD11b^+^Gr-1^high^Ly-6C^low^ subsets from mice bone marrow were sorted and treated with the PLGA/OVA NPs, followed by co-culture with the carboxyfluorescein succinimidyl ester (CFSE)-labelled OT-I CD8^+^ cells. Co-culture of OT-I CD8^+^ T cells with PLGA/OVA NPs-primed CD11b^+^Gr-1^+^ subsets upregulated the expression of IL-2, TNF-α, INF-γ, granzyme B, and perforin, resulting in proliferation of CD8^+^ T cells and differentiation into effector cytotoxic T lymphocytes (CTLs). *In vivo* proliferation of CFSE-labelled OT-I CD8^+^ cells in response to OVA was also obtained in the animals immunized with PLGA/OVA NPs. The results presented in this study demonstrate the ability of polymeric NPs to recruit two CD11b^+^Gr^−^1^+^ myeloid subsets for effective presentation of exogenous antigen to OT-I CD8^+^ T cells in the context of major histocompatibility complex (MHC) class I, leading to an induction of antigen-specific cell proliferation and differentiation into effector cells.

Biodegradable polymeric micro- or nano-particles (NPs) are of great interest in the field of drug delivery and have been extensively studied in vaccine delivery for the enhancement of presentation of exogenous antigens^1^,^2^,^3^,^4^,^5^,^6^, a process referred to as cross-presentation or cross-priming, in which the antigenic fragment derived from exogenous proteins is bound to the major histocompatibility complex (MHC) class I molecules of the antigen presenting cells (APCs) to stimulate the CD8^+^ T immune response^7^,^8^,^9^. The induction of cytotoxic CD8^+^ T cell-mediated immunity plays a pivotal role in the development of immunotherapeutic strategies against infection and cancer. Dendritic cells (DCs), the professional APCs in the processing and presentation of exogenous antigens, have served as the major target cells for antigen delivery to enhance vaccine efficacy^10^,^11^,^12^,^13^,^14^. Although it was reported in earlier studies that particulate antigens can promote presentation of the associated antigens to T cells via both macrophage and non-macrophage APCs that phagocytose the particles^15^, the delivery of antigens by nanoparticles (NPs) to other APCs for the elicitation of MHC class I immunity unfortunately has been largely ignored. The ability of neutrophils to process the phagocytosed bacteria via the MHC Class I pathway to trigger the CD8^+^ T cell responses and their ability to stimulate *in vitro* cross presentation of exogenous antigens employing the B3Z model have been previously reported^16^,^17^. Our recent study also demonstrated the activation of CD8^+^ T cells by the nanoparticles-primed Gr-1^high^ cells^18^. These results prompted us to further evaluate the potential of granulocytes from murine bone marrow to induce activation of cytotoxic T lymphocyte (CTL) effectors in nanoparticle (NPs)-based vaccination.

Immature myeloid cells in the bone marrow (BM) are a heterogeneous population of cells that differentiate into protective cell types such as granulocytes and macrophages^19^. BM granulocytes can be phenotypically characterized by the expression of the surface proteins CD11b and Gr-1, including the two isoforms Ly6C and Ly6G^19^,^20^. The CD11b^+^Gr-1^+^ subset is a heterogeneous myeloid population comprising at least two subsets: polymorphonuclear (PMN) and monocytic cells^21^. The polymorphonuclear granulocytes are the most abundant leukocytes continuously released from bone marrow (BM) into the blood circulation, and they play a critical role in innate immunity. Despite the established phagocytic activity of granulocytes, the role of BM CD11b^+^Gr-1^+^ cells in MHC class I antigen processing and presentation via polymeric nanoparticles (NPs) has been ignored.

In this study, we employed the anti-Gr-1 monoclonal antibody (RB6–8C5), previously used to detect the granulocyte-differentiation antigen on more differentiated granulocytes^22^, to characterize the two subsets of BM myeloid subsets, including the CD11b^+^Gr-1^high^Ly-6C^low^ (abbreviated as Gr-1^high^) subset that exhibits a polymorphonuclear or band-shaped nuclear morphology and the CD11b^+^Gr-1^low^Ly-6C^high^ (abbreviated as Gr-1^low^) subset, with a mononuclear morphology. We attempted to elucidate the role of CD11b^+^Gr-1^+^ polymorphonuclear (PMN) granulocytes in antigen cross presentation after treatment with the nanoparticle-based antigens. The CD8^+^ T cells from OT-I mice, expressing the transgenic T cell receptor (TCR) specific for OVA peptide residues 257–264 in the context of H2K^b^, were used to assess the effects of PLGA/OVA NPs on the activation of the OVA-specific CD8^+^ T cell response and the induction of the cytotoxic lymphocyte (CTL) effect. It was assumed that upon activation by the polymeric NPs-primed CD11b^+^Gr-1^+^ granulocytes, the antigen-specific CD8^+^ T cells undergo proliferation and differentiation into effectors (clonal expansion) that recognize specific peptides on MHC class I complexes and express type 1 cytokines, such as IFN-γ, TNF-α, and IL-2, for the elicitation of cytotoxicity (target elimination)^23^,^24^. The cytotoxic T lymphocytes (CTLs) are effector lymphocytes that play important roles in defence immunity against infectious diseases and cancers, in which perforin and granzyme B are involved in the induction of cell death, contributing to an efficient generation of immune effectors in the antigen specific immune response^25^.

The results of this study illustrated that priming the Gr-1^high^ and Gr-1^low^ subsets of BM CD11b^+^Gr-1^+^ cells with the PLGA/OVA NPs induced the expansion and proliferation of OVA-specific OT-I CD8^+^ T cells, resulting in an antigen-specific immune response in the context of MHC class I complexes.

## Results

We investigated the potential recruitment of polymorphonuclear (PMN) granulocytes from the mouse bone marrow (BM) by polymeric nanoparticles (NPs) for cross-presentation of exogenous antigens and stimulation of cytotoxic T lymphocyte (CTL) effector functions.

### Scanning electron microscopy (SEM) of PLGA/OVA NPs

**Figure 1:**
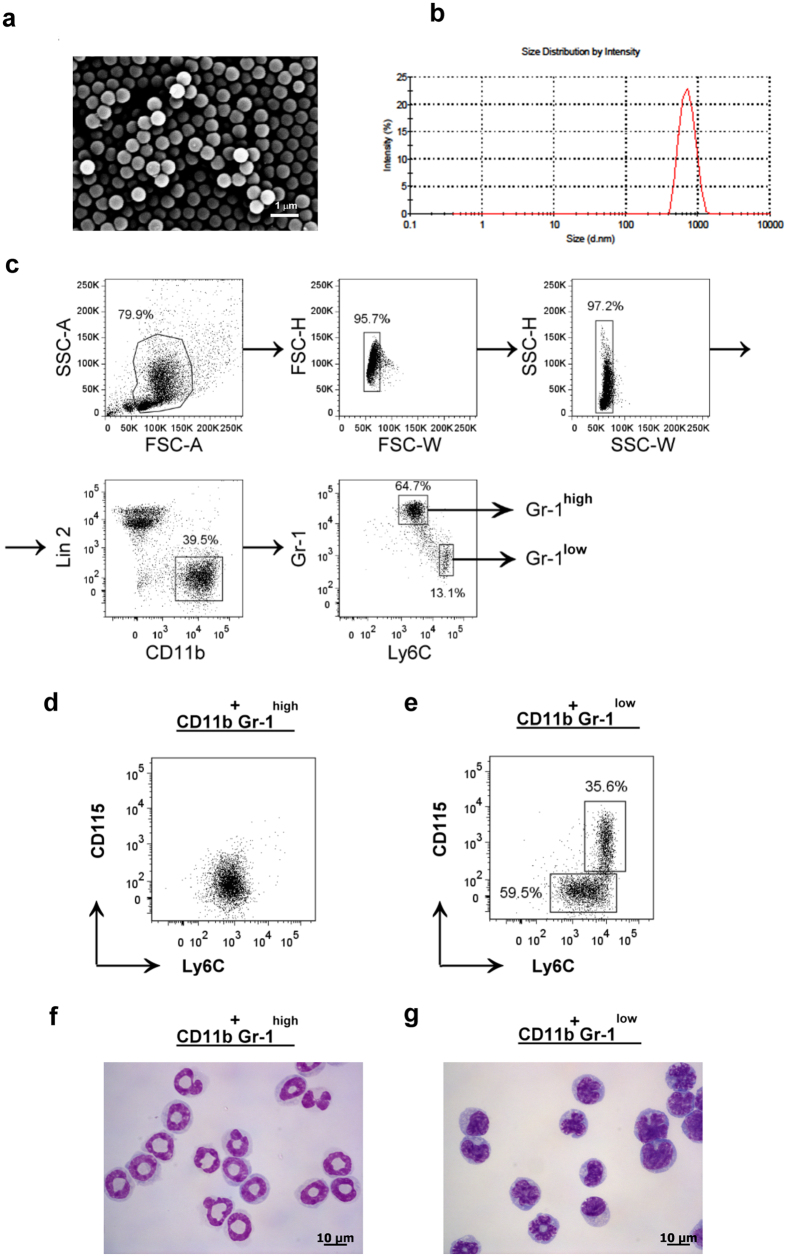
Scanning microscopic examination of PLGA/OVA NPs and sorting of the Gr-1^+^ subsets from mouse bone marrow (**a**), SEM image of the PLGA/OVA NPs. Scale bar = 1 μm. (**b**) Size distribution of the PLGA/OVA NPs. (**c**), Illustration of the gating scheme of Gr-1^high^ and Gr-1^low^ subsets from mouse bone marrow. Bone marrow cells were first gated on single live cells and sorted by flow cytometry based on their expression of Lin2 (CD3/CD19/B220/Ter119/NK1.1), CD11b, Ly-6C, and Gr-1. (**d**,**e**) Dot plots displaying the heterogeneity of the Gr-1^low^ subset that partly express CD115, whereas Gr-1^high^ cells do not. Figures (**f**,**g**) illustrate the microscopic morphology of the CD11b^+^Gr-1^high^ (**f**) and CD11b^+^Gr-1^low^ (**g**) subsets after staining with the Wright-Giemsa stain. Scale bar = 10 μm.

The electron micrograph of the PLGA/OVA NPs, prepared by the double emulsion and solvent evaporation method, is shown in Fig. 1a, illustrating the spherical shape with a mean particle size of 690 ± 7.1 nm. Physicochemical characterization of the PLGA/OVA NPs and the release profile of antigen from PLGA/OVA NPs have been previously reported^18^. The mean particle size of the PLGA/OVA NPs was 690 ± 7.1 nm, and the size distribution is shown in Fig. 1b. The zeta potential of the PLGA/OVA NPs, determined by a Malvern Zetasizer Nano ZS, was −30.5 ± 0.5 mV, and the encapsulation efficiency (the ratio of the amount of ovalbumin in the NPs to the initial amount loaded) was approximately 52.7%^18^.

### Sorting of Lineage2^−^CD11b^+^Gr-1^high^ and Lineage2^−^CD11b^+^Gr-1^low^ subsets by fluorescence-activated cell sorting (FACS) and microscopic examination

Bone marrow (BM) cells were flushed and isolated from the femurs and tibias of mice aged 6–8 weeks. The Lin2^−^CD11b^+^Gr-1^high^Ly-6C^low^ (Gr-1^high^) and Lin2^−^CD11b^+^Gr-1^low^Ly-6C^high^ (Gr-1^low^) subsets were sorted using a FACSAria III (BD Biosciences, San Jose, CA) (Lin2: CD3/CD19/B220/Ter119/NK1.1)^18^,^26^, and the morphology of the sorted populations was assessed by Wright-Giemsa staining. The gating scheme for sorting of these two subpopulations is presented in Fig. 1c. The phenotypic analysis by flow cytometry showed that Gr-1^high^ cells are Ly-6C^low^ and polymorphonuclear in morphology, whereas most Gr-1^low^ cells are Ly-6C^high^ and mononuclear with a heterogeneous nuclear shape. In contrast to the expression of CD115 in 35.6% of the Gr-1^low^ subset, most Gr-1^high^ cells do not express CD115 (Fig. 1c–g).

### Proliferation and expression of the activation markers CD25 and CD69 in OT-I CD8^+^ T cells after co-culture with PLGA/OVA NPs-primed Gr-1^+^ cells

**Figure 2:**
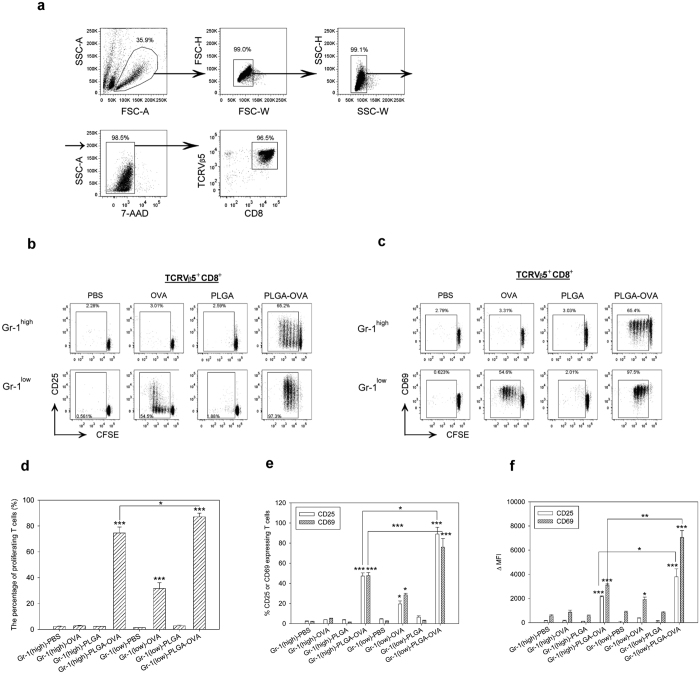
PLGA/OVA NPs primed Gr-1^high^ and Gr-1^low^ subsets from the murine bone marrow by the PLGA/OVA NPs induced cross-presentation of antigens in OT-I CD8^+^ T-cells. The representative flow cytometric plots, illustrating (**a**), the gating scheme of OT-I TCRVβ5^+^CD8^+^ T-cells after co-culture with PBS, OVA, PLGA NPs, or PLGA/OVA NPs -treated Gr-1^+^ subset and (**b**,**c**), progressive cell division of CFSE-labelled OT-I CD8^+^ T cells in response to NPs treatment. Proliferation and the percentages of CD25^+^ and CD69^+^ T cells after the treatment are shown in (**d**,**e**); (**f**) illustrates the delta mean fluorescence intensities (ΔMFI) of CD25 and CD69 in the gated OT-I CD8^+^ T cells, which are presented as the mean values of four separate experiments relative to the isotype controls. Asterisks denote statistically significant differences *vs*. the PBS control in the same group (*p < 0.05; **p < 0.01; ***p < 0.001; by one-way ANOVA).

To examine the effect of Gr-1^+^ cells after pre-treatment with the polymeric nanoparticles on the proliferation of OT-I CD8^+^ T cells *in vitro*, CD8^+^TCRVβ5^+^ T cells were enriched from the spleens of OT-I mice by negative selection. The purity of the sorted fraction was approximately 96.5% (Fig. 2a). These cells were then labelled with carboxyfluorescein succinimidyl ester (CFSE) and co-cultured for 72 hrs with the sorted Gr-1^+^ cells, either the Gr-1^high^ or Gr-1^low^ subset, that were pre-treated for 16 hrs with PBS, OVA, PLGA NPs, or PLGA/OVA NPs. The OT-I CD8^+^ T cell response, assessed by the CFSE-dilution assay, showed an extensive cell division and proliferation in response to the treatment with PLGA/OVA NPs compared to the control groups treated with PBS, OVA, or PLGA NPs alone (Fig. 2b–f).

CD25, the α chain of the IL-2 receptor, and CD69 are T cell activation markers. To determine if the Gr-1^+^ cells primed with PLGA/OVA NPs induced CD 25 and CD69 expression on T cells, the cells were surface stained with fluorochrome-conjugated antibodies against CD25 and CD69. Figure 2b–f illustrate a significant proliferation and upregulation of the expression of CD25 and CD69 in the TCRVβ5^+^CD8^+^ T cells after co-culture with the PLGA/OVA NPs-treated Gr-1^high^ or Gr-1^low^ subsets compared to the control groups treated with PBS, OVA, or PLGA NPs without OVA, which did not induce significant CD25/CD69 expression or cell proliferation (Fig. 2b–f). Statistical analysis revealed a significant induction of cell proliferation and more pronounced activation phenotypes in OT-I CD8^+^ T cells when co-cultured with the PLGA/OVA NPs-primed Gr-1^low^ subset compared to the Gr-1^high^ group (Fig. 2d–f). These data demonstrated the antigen presentation capabilities of the Gr-1^+^ cells, including both the Gr-1^high^ or Gr-1^low^ subsets, after acquiring antigens from the PLGA/OVA NPs.

### Surface expression of F4/80, CD11c, CD115, and I-A/I-E in Gr-1^high^ and Gr-1^low^ subsets

**Figure 3:**
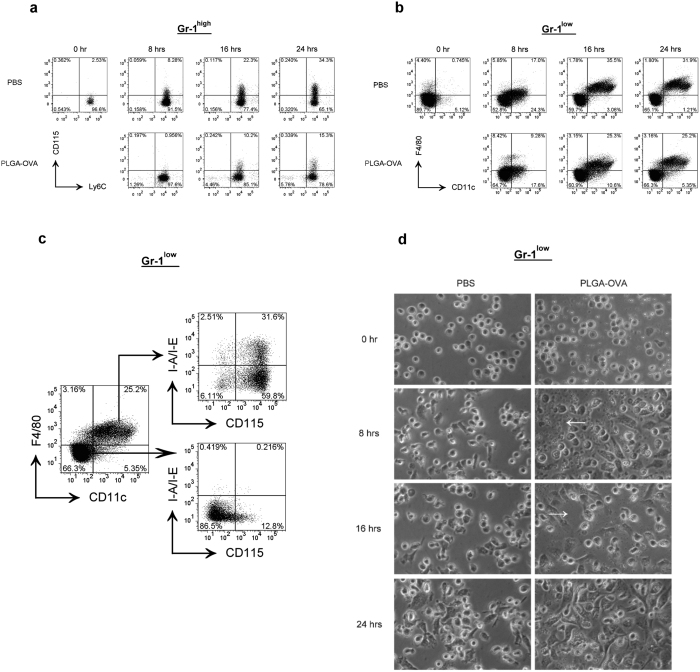
Time-course of surface expression of Ly-6C, CD115, CD11c, F4/80, and I-A/I-E in the Gr-1^high^ and Gr-1^low^ subsets after treatment with the PLGA/OVA NPs. (**a**) Gr-1^high^ and (**b**), Gr-1^low^ cells were treated with PLGA/OVA NPs in complete RPMI 1640 medium containing 5 ng/ml GM-CSF, and incubated at 37 °C for 0, 8, 16, and 24 hrs, followed by flow cytometric analysis. (**c**) The expression of I-A/I-E and CD115 in the DP (CD11c^+^F4/80^+^) and DN (CD11c¯F4/80¯) populations at 24 hrs after treatment of Gr-1^low^ cells with the PLGA/OVA NPs. (**d**) Visual examination of the Gr-1^low^ cells after treatment with the PLGA/OVA NPs with a phase contrast microscope and a digital camera. The presence of PLGA/OVA NPs can still be observed at 8 hrs after incubation (arrow head), but these NPs were cleared up at 16 hrs (arrow head) and extensively ingested at 24 hrs post-incubation when the spindle-shaped cells became abundant.

Because these cells cannot survive *in vitro* without cytokines, such as granulocyte-macrophage colony-stimulating factor (GM-CSF), to examine the effect of the treatment of PLGA/OVA NPs on the phenotypic changes of Gr-1^+^ cells, the Gr-1^high^ or Gr-1^low^ subsets were placed in 96-well plates at 1 × 10^6^ cells/ml in complete RPMI 1640 medium containing 5 ng/ml GM-CSF, followed by treatment with PBS, 100 μg/ml PLGA NPs, or PLGA/OVA NPs for 0, 8, 16, or 24 hrs. The cells were then harvested, washed with PBS, and analysed by flow cytometry. The data in Fig. 3 show the induction of the expression of CD115, a receptor for macrophage colony stimulating factor (M-CSF), in the Gr-1^high^ population. Treatment of Gr-1^high^ cells with the PLGA/OVA NPs reduced CD115 expression compared to the PBS control (Fig. 3a).

Approximately 25–35% of Gr-1^low^ cells expressed high levels of CD11b but were negative in CD11c and F4/80, the dendritic cells and macrophage markers, started expressing both markers after 16 hrs incubation in the culture medium containing GM-CSF (Fig. 3b). Approximately 34% of cells in the F4/80^+^CD11c^+^ double-positive (DP) subset also express MHC II, and 13% of cells in the F4/80^−^CD11c^−^ double-negative (DN) subset express CD115 (Fig. 3c). Plasticity has long been recognized as a hallmark in the differentiation pathway of myelomonocytic cells. These data demonstrate that less than 35% of Gr-1^low^ cells, but not the Gr-1^high^ subset, differentiated into F4/80^+^CD11c^+^ mononuclear phagocytes^18^,^27^, exhibiting characteristics of macrophages and dendritic cells, and that *in vitro* cell transformation induced by the polymeric NPs was relatively insignificant.

Microscopic examination of Gr-1^low^ cells illustrated that in comparison to the PBS treated control cells, treatment with PLGA/OVA NPs for 8 hrs induced the formation of spindle-shaped cells that are attached to the bottom of the plates, whereas the PLGA/OVA NPs are still present (Fig. 3d). These nanoparticles began to be cleared after incubation for 16 hrs when Gr-1^low^ cells start expressing CD11c and F4/80 (Fig. 3b and d), and were extensively ingested when the spindle-shaped cells became more abundant, compared to the PBS control, at 24 hrs post-treatment (Fig. 3d).

### Treatment of Gr-1^+^ cells with PLGA/OVA NPs enhanced intracellular cytokine expression

**Figure 4:**
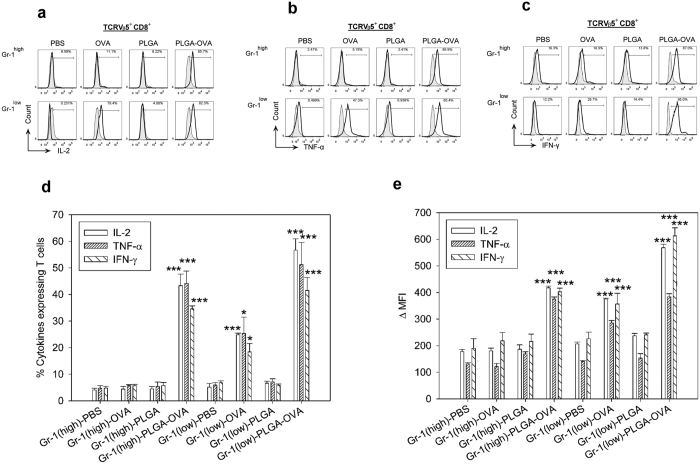
PLGA/OVA NPs-treated CD11b^+^Gr-1^+^ cells efficiently induced the expression of IL-2, TNF-α, and IFN-γ in the OT-I CD8^+^ T cells, as determined by intracellular cytokine (ICC) staining. Intracellular cytokine and surface staining by flow cytometric analysis in the PLGA/OVA NPs-treated CD11b^+^Gr-1^+^ cells, showed the enhanced expression of (**a**), IL-2, (**b**), TNF-α, and (**c**), IFN-γ. The grey and tinted area represents the isotypes. (**d**,**e**) Statistical analysis of the percentages of cytokine-expressing T cells and the delta mean fluorescence intensities (ΔMFI) of OT-I CD8^+^ T cells relative to the isotype controls, performed by one-way ANOVA. Data are displayed as the mean ± standard error mean (SEM) from five separate experiments. Asterisks indicate statistically significant differences *vs*. the PBS control in the same group (*p < 0.05; ***p < 0.001; by one-way ANOVA).

To assess the cytokine secretion induced by the treatment of PLGA/OVA NPs, the Gr-1^high^ or Gr-1^low^ cells were pre-treated for 16 hrs with PLGA NPs, with or without the OVA antigen, followed by co-culture for 72 hrs with CD8^+^ T cells isolated and negatively enriched from the spleens of OT-I mice. The cells were then stained with fluorochrome-conjugated mAbs against CD8α, TCRVβ5.1, Gr-1, Ly-6C, and CD11b. Co-culture of OT-I CD8^+^ cells with Gr-1^+^ cells pre-treated with PLGA/OVA NPs induced the expression of Th1-like cytokines, such as IL-2, TNF-α, and IFN-γ, at significantly higher levels than the control groups treated with PBS, OVA, or PLGA NPs alone (Fig. 4a–c). The percentages of cytokine-secreting cells were analysed and compared in the gated CD8^+^TCRVβ5^+^ T cells. Selected data are presented as delta mean fluorescence intensity (ΔMFI); *i.e*., the MFI of cytokine expression in the CD8^+^TCRVβ5^+^ T cells in each group minus the corresponding cytokine levels of CD8^+^TCRVβ5^+^ T cells in the matched isotypes. The data in the bar graphs (Fig. 4d–e) depicting the percentages and the ΔMFI of cytokine-expressing cells demonstrate that treatment of CD8^+^TCRVβ5^+^ T cells with the Gr-1^+^ subsets pre-treated with the PLGA/OVA NPs significantly upregulated the expression of Th1-like cytokines in the OT-I CD8^+^ T cells.

### Detection of perforin and granzyme B by flow cytometry and fluorescence microscopy

**Figure 5:**
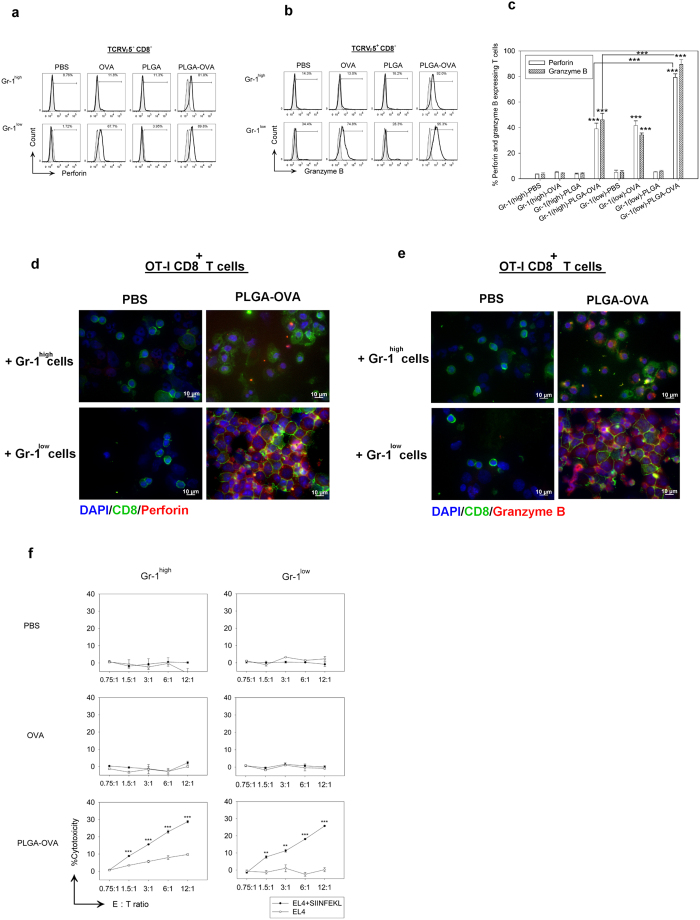
PLGA/OVA NPs-treated Gr-1^high^ or Gr-1^low^ subsets induced the expression of perforin and granzyme B in the OT-I CD8^+^TCRVβ5^+^ T cells, resulting in the cytotoxic lymphocyte (CTL) effect. Intracellular and surface staining and analysis by flow cytometry shows the expression of (**a**) perforin and (**b**) granzyme B in the OT-I CD8^+^TCRVβ5^+^ T cells, after co-culture with PBS, OVA, PLGA NPs, or PLGA/OVA NPs-treated Gr-1^high^ or Gr-1^low^ subsets. Grey and tinted area are isotype controls. (**c**), The data, presented as the mean ± standard error mean (SEM) of five separate experiments, showed the increased percentages of perforin and granzyme B-expressing cells, relative to the isotype controls, in the OT-I CD8^+^ population after co-culture with the CD11b^+^Gr-1^+^ subsets, pre-treated with PBS, OVA, PLGA NPs, or PLGA/OVA NPs. Asterisks denote statistically significant differences *vs*. the PBS control in the same group (*p < 0.05; ***p < 0.001; by one-way ANOVA). (**d**,**e**) Fluorescence microscopic examination of CD8α^+^TCRVα2^+^ T cells, after co-culture with PBS or PLGA/OVA NPs-treated Gr-1^high^ or Gr-1^low^ subsets, confirmed the expression of perforin (**d**) and granzyme B (**e**). (**f**) PLGA/OVA NPs-primed Gr-1^+^ cells induced cytotoxic T-lymphocyte (CTL) activity of the OT-I CD8^+^ T cells. Lysis of the target cells was determined using the LDH release assay. The values presented are the mean values ± SEM of triplicate experiments. Asterisks indicate statistically significant differences between the SIINFEKL-pulsed EL4 cells and the untreated controls at the same E:T ratio (**p < 0.01; ***p < 0.001; by t-test).

Acquisition of the effector properties, such as upregulation of perforin and granzymes, is critical in the generation of cytotoxic T lymphocytes (CTLs). To detect the expression of granzyme B and perforin in the secretary granules of OT-I CD8^+^ cytotoxic T lymphocytes (CTLs) after co-culture with the PLGA/OVA NPs primed Gr-1^+^ populations, cells were stained with mAbs against granzyme B and perforin and analysed with a BD FACSVerse cytometer. The expression of these cytolytic effector molecules was significantly upregulated in the PLGA/OVA NPs-treated group compared to the control groups treated with PBS, OVA, or PLGA NPs alone (Fig. 5a–c). This finding is substantiated with further examination by fluorescence microscopy, confirming that treatment of Gr-1^+^ cells, either the Gr-1^high^ or Gr-1^low^ subset, with the PLGA/OVA NPs induced significant upregulation of perforin and granzyme B in the OT-I CD8^+^ T cells, and that the expression was relatively higher when co-cultured with the Gr-1^low^ cells than those with the Gr-1^high^ subset (Fig. 5d–e).

### Treatment of Gr-1^+^ cells with PLGA/OVA NPs induced the cytolytic activity of OT-I CD8^+^ T cells

To determine the effect of PLGA/OVA NPs on cytolytic activity, the enriched OT-I CD8^+^ T cells were co-cultured for 72 hrs with the Gr-1^high^ or Gr-1^low^ subsets that were pre-treated for 16 hrs with PBS, OVA, PLGA NPs, or PLGA/OVA NPs. The cells were then co-cultured for 4 hrs with EL4 cells pulsed with or without 1 μM SIINFEKL peptide followed by cytolytic assay using the CytoTox96 non-radioactive cytotoxicity assay kit (Promega). The results in Fig. 5f demonstrate that co-culture of OT-I CD8^+^ cells with PLGA/OVA NPs-primed Gr-1^+^ cells, either the Gr-1^high^ or Gr-1^low^ subset, stimulated antigen-specific cytotoxic activity in the SIINFEKL-pulsed target EL4 cells, in contrast to the untreated EL4 control groups (Fig. 5f).

### Enhanced production of OVA-specific IgG in the blood of animals after immunization with PLGA/OVA NPs

**Figure 6:**
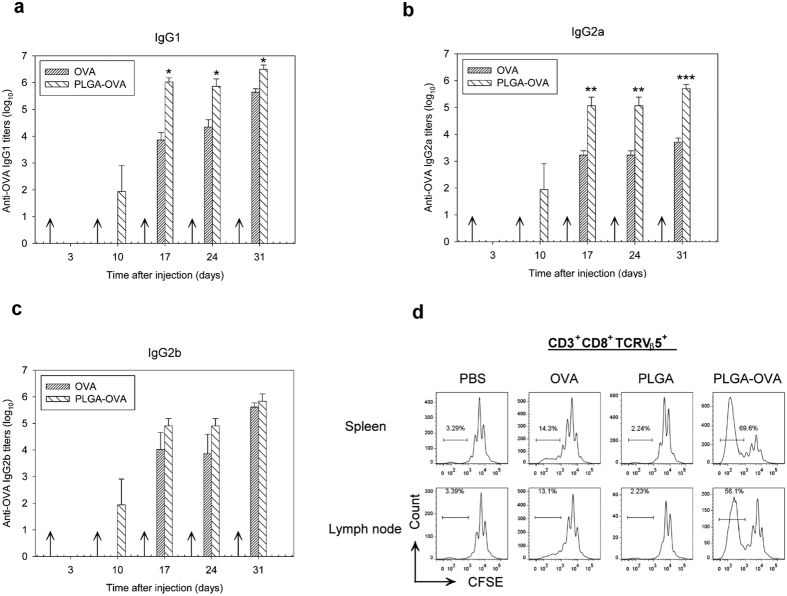
Immunization of mice with PLGA/OVA NPs increased the titres of anti-OVA antibody and induced proliferation of OT-I CD8^+^ T cells *in vivo*. (**a**) Antibody titres are expressed as the dilution factor giving OD 450 values above 0.1. The data represent mean titres ± SEM of (**a**), IgG1, (**b**) IgG2a, and (**c**), IgG2b from three independent experiments. Statistically significant differences between the PLGA/OVA NPs-treated group *vs*. the OVA-control were denoted with asterisks (*p < 0.05; **p < 0.01; ***p < 0.001; by t-test). (**d**) The proliferation of adoptively transferred OT-I CD3^+^CD8^+^TCRβ5^+^ cells, isolated from spleen and lymph nodes of the recipient mice that were pre-treated four days earlier with PBS, OVA, PLGA NPs, or PLGA/OVA NPs, was analysed by flow cytometry. The gating in the graphs shows the percentage of the proliferating OT-I CD8^+^ cells. Data presented are representative of three independent experiments.

In an attempt to evaluate the impact of PLGA/OVA NPs on the induction of the humoral immune response *in vivo*, C57BL/6J mice were *i.v*. injected with PBS, OVA, PLGA NPs, or PLGA/OVA NPs on days 0, 7, 14, 21, and 28, followed by blood collection on days 3, 10, 17, 24, and 31. The serum antibody titres were quantified by enzyme-linked immunosorbent assay (ELISA) with peroxidase-conjugated rat anti-mouse IgG1, IgG2a, or IgG2b and the plates were read with a spectrophotometer. The OVA-specific antibody response in each immunization group is shown in Fig. 6a–c. The OVA-specific IgG levels in the control groups treated with PBS and PLGA NPs were not detectable (data not shown), whereas mice immunized with PLGA/OVA generated significantly higher titres of IgG isotypes, including IgG1 and IgG2a, than the group immunized with OVA alone (Fig. 6a–c).

### *In vivo* proliferation of antigen-specific OT-I CD8^+^ T cells induced by the PLGA/OVA NPs

To monitor the antigen-specific T cell proliferation, the CD8^+^ T cells were isolated and enriched from spleens of OT-I mice, labelled with CFSE and *i.v*. injected into the tail veins of irradiated C57BL/6J recipient mice. Twenty-four hrs later, the mice were *i.v*. injected with PBS, OVA, PLGA NPs, or PLGA/OVA NPs and euthanized at 4 days post-treatment. Cells from the spleen and lymph nodes were analysed by flow cytometry. The proliferation profiles of the OT-I CD8^+^ T cells, gated on the 7-AAD¯CD3^+^CD8^+^TCRVβ5^+^ population, are shown in Fig. 6d, demonstrating a significant proliferation of the OT-I CD8^+^ T cells in the recipient mice immunized with PLGA/OVA NPs, compared to the control groups treated with PBS, OVA, or PLGA NPs alone (Fig. 6d).

## Discussion

In view of the emergence of nanotechnology and the potential application of polymeric nanoparticles in cancer vaccines, we investigated in this study the combined potential of polymeric nanoparticles and the phagocytic capability of bone marrow Gr-1^+^ granulocytes, including the Gr-1^high^ and Gr-1^low^ subsets, for cross presentation of exogenous antigens to boost the MHC class I immune response. Treatment of BM Gr-1^+^ cells, either the Gr-1^high^ or Gr-1^low^ subsets, with antigen-encapsulated polymeric nanoparticles (NPs) significantly stimulated the *in vitro* proliferation of antigen-specific CD8^+^ T cells and enhanced the secretion of Th1 cytokines, such as IL-2, TNF-α, and IFN-γ, compared to the control groups treated with free antigen or the empty PLGA NPs alone (Fig. 4). It is noteworthy that these PLGA/OVA NPs do not stimulate the maturation of the costimulatory molecules in the bone marrow-derived dendritic cells (BMDCs) (data not shown). In contrast to the Gr-1^low^ group, Gr-1^high^ cells do not simulate significant expression of these cytokines in OVA-treated OT-I CD8^+^ cells (Fig. 4). Cytometric analysis of Gr-1^high^ cells with a monoclonal antibody 25-D1.16 (eBioscience) specific for the OVA (257–264) peptide SIINFEKL bound to H-2K^b^ of MHC class I showed the capability of cross-presentation of the H-2K^b^ bound OVA-peptide in the context of MHC I when treated with the PLGA/OVA NPs compared to the control groups^18^. It is established that monocytes can differentiate into DCs when receiving a phagocytic stimulus^27^. Upon *in vitro* culture in the presence of GM-CSF, the expression of CD115 in CD11b^+^Gr-1^low^ cells was upregulated, and approximately 30–35% of CD11b^+^Gr-1^low^ cells became CD11c^+^F4/80^+^ double-positive (DP), whereas approximately over 65% of the population remained CD11c^−^F4/80^−^ double-negative (DN) (Fig. 3b). In the double-positive (DP) population, 90% of cells express CD115 (CSFR), indicating the trans-differentiation potential of these cells into dendritic cells or macrophage-like monocytic phagocytes in the presence of GM-CSF, regardless of the treatment with the PLGA/OVA NPs. The CD11c^+^F4/80^+^ double-positive (DP) population can be further subdivided into MHC II^+^ and MHC II¯ granulocytic fractions. The CD11c¯F4/80¯ double-negative (DN) population, however, is both CSFR¯ and MHC II¯(Fig. 3c). Phagocytosis of the PLGA NPs by these two subsets, DP and DN, did not induce significant phenotypic changes in the cells. Gr-1^low^ cells exhibited superior capabilities of cross-presentation compared to the Gr-1^high^ subset (Fig. 2b–f), presumably caused by the trans-differentiation of 25–35% of Gr-1^low^ population into CD11c^+^ dendritic cells, the most potent antigen presenting cells, in the culture medium containing GM-CSF.

The expression of CD11b and Gr-1 has been associated with immunosuppression in cancer or infection in the form of myeloid-derived suppressor cells (MDSCs). In contrast to the previous report by Peranzoni *et al*.^21^, employing the anti-CD11b and anti-Gr-1 mAb (RB6-8C5) mAbs to distinguish the two murine myeloid subsets of myeloid derived suppressor cells (MDSCs) with immunosuppressive properties^21^, including the CD11b^+^Gr-1^high^Ly6G^+^Ly6C^low/int^ (polymorphonuclear) and CD11b^+^Gr-1^int^Ly6G^−^Ly6C^high^ (mononuclear) cells, the results obtained in this study demonstrated that treatment of these cells with the antigen-containing polymeric NPs resulted in immunostimulation rather than immunosuppression. The experimental evidence from this study also demonstrated the potential capability of the CD11b^+^Gr-1^+^ cells, including the Gr-1^high^ and the Gr-1^low^ subsets, to present exogenous antigens in the context of MHC class I to the CD8^+^ T cells when primed with the PLGA/OVA NPs, leading to the upregulation of type 1 cytokines (Fig. 4) and the acquisition of cytolytic molecules such as perforin and granzyme B (Fig. 5), a signature of CTL activation and effector function, to eliminate the pathogen-infected or oncoprotein-specific transformed cells, one of the most desired effect in the design of vaccine-based cancer therapy. To the best of our knowledge, results reported herein showed the first experimental evidence demonstrating the acquisition of antigen-specific cytolytic T effector functions via the polymeric NPs-primed Gr-1^+^ subsets. However, the data in Fig. 5 reveals that the Gr-1^low^ subset express higher levels of perforin and granzyme B and hence exhibit higher antigen presentation capability than the Gr-1^high^ subset, presumably due to the trans-differentiation of Gr-1^low^ cells into CD11c^+^ dendritic cells in the presence of GM-CSF. This speculation, on the other hand, was not explicitly justified in the results shown in Fig. 5f, where both the Gr-1^high^ and Gr-1^low^ subsets exhibit similar antigen-presentation capabilities on the stimulation of the CTL effect.

Our recent studies on cellular biodistribution of polymeric nanoparticles have illustrated the kinetics and localization of polymeric nanoparticles in the immune system after *i.v*. injection^28^. The *i.v*. administration route prompts the uptake of nanoparticles by the APCs in the circulation faster than any other routes, resulting in accumulation of nanoparticles in the lymphoid organs such as spleen and bone marrow^29^. The nanoparticles can then be taken up by the phagocytic APCs, such as dendritic cells and macrophages, followed by antigen cross-presentation and the ensuing antigen-specific immune response. The *in vivo* expansion of adoptively transferred CFSE-labelled OT-I CD8^+^ T cells was confirmed in this study in the recipient mice immunized with the PLGA-NPs (Fig. 6d), suggesting that the PLGA/OVA NPs are able to stimulate the antigen-specific CD8^+^ T cell proliferation in the PLGA/OVA NPs immunized animals. The function of polymeric nanoparticle-mediated antigen delivery in the cross-presentation of exogenous antigens *in vivo* is thus confirmed.

## Conclusions

We have demonstrated in this study the efficient recruitment of Gr-1^+^ subpopulations, including both the Gr-1^high^ and Gr-1^low^ subsets from murine bone marrow, by PLGA/OVA nanoparticles (NPs) for cross presentation of exogenous antigens in the context of MHC class I complexes, resulting in antigen-specific OT-I CD8^+^ T cell proliferation and upregulation of the expression of Th1-type cytokines, including IL-2, TNF-α, IFN-γ, and effector molecules such as perforin and granzymes B, leading to the effector T cell response and antigen-specific cytotoxic T-lymphocyte (CTL) effects. The experimental results obtained in this study on nanoparticulate antigens have a crucial implication for the future design of vaccine delivery.

## Materials and Methods

### Materials

PURASORB PDLG (DL-lactide and glycolide) 5004A (50/50 DL-lactide/glycolide copolymer, MW44000), a biodegradable poly(lactic-co -glycolic acid) (PLGA) copolymer, was kindly provided by Purac Biomaterials (Gorinchem, The Netherlands). Polyvinyl alcohol (MW15,000) was obtained from Fluka and the OVA peptide 257–264 (SIINFEKL) was obtained from PolyPeptide Group (Strasbourg, France). Fetal bovine serum (FBS) and RPMI 1640 cell culture medium were obtained from HyClone Laboratory and Gibco Inc. respectively. 5-(and-6)-carboxyfluorescein diacetate succinimidyl ester (CFSE) was obtained from Molecular Probes. 4′,6-Diamidino-2-phenylindole dihydrochloride (DAPI) was purchased from Sigma-Aldrich (St. Louis, MO). The CytoTox 96 Non-Radioactive Cytotoxicity Assay was purchased from Promega (Madison, WI).

### Antibodies

Most fluorochrome-conjugated antibodies, unless otherwise indicated, were purchased from Biolegend (San Diego, CA) or eBioscience (San Diego, CA), including: CD3 (17A2), CD8 (53–6.7), CD11b (M1/70), CD11c (N418), CD16 and CD32/Fc block (93), CD19 (1D3), CD45R/B220 (RA3-6B2), CD115/CSF-1R (AFS98), Gr-1 (RB6-8C5), Ly-6C (HK1.4), MHC-II (M5/114.15.2), NK1.1 (PK136), Ter 119 (TER-119), TCRVα2 (B20.1), and TCRVβ5.1 (MR9-4). Streptavidin–PE-Cy7, streptavidin–PerCP–Cy5.5, and 7-amino-actinomycin D (7-AAD) viability staining solutions were obtained from eBioscience, and streptavidin-APC-Cy7 was obtained from Biolegend. ExtrAvidin−TRITC was obtained from Sigma-Aldrich (St. Louis, MO).

### Cell lines

EL4 cells, an H-2^b^ restricted ovalbumin (OVA)-expressing murine thymoma cell line, were maintained in Dulbecco’s modified Eagle’s medium (DMEM) supplemented with 100 μg/ml streptomycin, 100 U/ml penicillin, 4 mM L-glutamine, and 10% foetal bovine serum. EG7 cells, the OVA-expressing EL4 line, were maintained in complete DMEM containing 10 mM HEPES and 1.0 mM sodium pyruvate supplemented with 50 μM β-mercaptoethanol, 400 μg/ml G418, and 10% FBS.

### Animals

Adult six- to ten-week old C57BL/6J mice and C57BL/6J-background OT-I mice were bred and housed in the animal care facility of our university. The OT-I transgenic mice, containing the transgenic inserts for mouse TCRα-V2 and TCRβ-V5 genes, express an MHC class I-restricted TCR specific for ovalbumin (OVA) peptide residues 257–264 (SIINFEKL) in the context of H2K^b^. All experiments involved in animals were performed in accordance with relevant guidelines for the use of laboratory animals by the National Research Council USA, and the protocols were reviewed and approved by the Animal Care and Use Committee of National Taiwan University Medical College.

### Preparation of PLGA and PLGA//OVA nanoparticles

PLGA and PLGA/OVA NPs, the PLGA NPs containing the OVA antigen, were prepared by the water-in-oil-in-water (w/o/w) double emulsion and solvent evaporation method, as previously described^18^. A small amount of lyophilized NPs was placed on a double-sided tape attached on a sample stand and coated with a thin layer of gold using a Hitachi E-1010 ion sputter, and the surface morphology of the PLGA/OVA nanoparticles was examined by scanning electron microscopy (SEM) with a Hitachi S-4800 scanning electron microscope.

### Enrichment and purification of OT-I CD8^+^ T-cells by immunomagnetic depletion

CD8^+^ T cells were isolated from the spleens of OT-I transgenic mice and enriched by negative selection. Briefly, the spleens were isolated and prepared from C57BL/6J mice by disruption of spleens using frosted glass slides in complete RPMI 1640 medium containing 10% FBS. The isolated cell suspensions were passed through a nylon mesh and suspended in RPMI 1640 medium, followed by treatment with the ACK (ammonium chloride-potassium) lysing buffer^30^. The cells were further enriched by negative selection with an mAb cocktail specific for CD11b (M1/70), CD19 (1D3), B220 (RA3-6B2), erythroid (TER-119), Gr-1 (RB6-6C5), CD4 (GK1.5), MHC class II (M5/114), and F4/80 (BM8). One times 10^8^ cells were immuno-reacted with 2 ml BioMag goat anti-rat IgG magnetic beads (Qiagen) at a 1:10 cell-to-bead ratio to deplete CD4^+^ lymphocytes, granulocytes, B cells, macrophages, and erythrocytes.

### Purification of bone marrow granulocytes by fluorescence-activated cell sorting (FACS)

Bone marrow cells were prepared from the femurs and tibias of C57BL/6J mice. Briefly, the mice were anesthetized with isoflurane and euthanized. Bone marrow cells were harvested by flushing cells from the tibias and femurs of adult C57BL/6J mice, followed by lysis of red blood cells with ACK (ammonium-chloride-potassium) lysing buffer containing 154.95 mM ammonium chloride, 9.99 mM potassium carbonate, and 0.0995 mM EDTA. For phenotyping, sorted cells were stained with 7-AAD and fluorochrome-conjugated rat anti-mouse mAbs against Lineage 2 (Lin2), CD11b, Gr-1, and Ly-6C, where Lin2 includes the antibodies against CD3/CD19/NK1.1/B220/Ter119^26^. The Lin2¯CD11b^+^ Gr-1^high^ and Lin2¯CD11b^+^ Gr-1^low^ subsets were sorted with a BD FACSAria III high-speed cell sorter (BD Biosciences) by the Cell Sorting Core Facility of our university. The purity of the final DC subpopulations was routinely greater than 98.5%.

### Phenotypic analysis by flow cytometry and examination of cell morphology by Wright-Giemsa staining and microscopy

Gr-1^high^ and Gr-1^low^ subsets, isolated from bone marrow of mice by FACS, were stained with fluorochrome-conjugated mAbs against using the antibodies against Lin2, CD11b, Gr-1, and Ly-6, and analysed by flow cytometry with a LSRFortessa flow cytometer (BD Biosciences).

To examine the morphology of these cells, the two subpopulations were suspended in the cell culture medium at 1 × 10^5^ in 200 μl PBS, and cytospun at 1,000 rpm for 3 min onto glass slides with a Cytospin 4 cytocentrifuge (Thermo Scientific). The cells were fixed with methanol for 10 min at room temperature, washed with PBS, and stained with Wright-Giemsa stain (Sigma-Aldrich) for 1 min at room temperature^31^. The slides were then washed with double distilled water and dried, followed by mounting with DPX mountant (Sigma). The cell morphology was examined with a Zeiss Axio Imager A1 microscope and photographed.

### Labelling of OT-I CD8^+^ T cells with carboxyfluorescein succinimidyl ester (CFSE)

The enriched fractions of OT-I CD8^+^ T cells were labelled with 5 μM CFSE at 37 °C for 10 min. Then, the cells were thoroughly washed with 1% FBS/PBS to remove the excess dye and used for both the *in vitro* or *in vivo* T cell proliferation and antigen presentation assays, as previously described^32^,^33^,^34^.

### OT-I CD8^+^ T cell proliferation on cross presentation

The CFSE-labelled OT-I CD8^+^ T cells (1 × 10^5^ cells per well) were co-cultured at a 1:1 ratio in U-bottomed 96-well plates with the myeloid cells from mice bone marrow, either the Lin2¯CD11b^+^Gr-1^high^ (Gr-1^high^) and Lin2¯CD11b^+^Gr-1^low^ (Gr-1^low^) subset, for 3 days in complete RPMI 1640 medium containing 10% FCS, 100 U/ml penicillin and 100 μg/ml streptomycin, 2 mM L-glutamine, 100 μM β-mercaptoethanol, 2 mM L-glutamine, l5 ng/ml GM-CSF, and 10% FBS. The division of the OT-I CD8^+^/TCR^+^ T cells was analysed with a BD FACSVerse™ flow cytometer (BD Biosciences), and the proliferating OT-I CD8^+^ T cells were identified by the numbers of 7-AAD^−^CD8^+^CFSE^low^ cells. Activation markers on viable CFSE^+^ OT-I populations were characterized by simultaneous staining of the cells with fluorochrome-conjugated rat anti-mouse CD8, TCRVβ5, CD25, and CD69 mAbs.

### Intracellular cytokine (ICC) staining and flow cytometric analysis of IL-2, IFN-γ, TNF-α, granzyme B, and perforin in OT-I CD8^+^ T cells

The sorted CD11b^+^Gr-1^+^ populations, including the Lin2^−^CD11b^+^Gr-1^high^Ly-6C^low^ (Gr-1^high^) granulocytes and Lin2^−^CD11b^+^Gr-1^low^Ly-6C^high^ (Gr-1^low^) monocytes, were sorted by a BD FACSAria III sorter and treated with PBS, OVA (100 μg/ml), 100 μg/ml PLGA NPs, or 100 μg/ml PLGA/OVA NPs. The cells were incubated at 37 °C for 16 hrs in complete RPMI 1640 medium containing 5 ng/ml GM-CSF, followed by co-culture for 72 hrs at a 1:1 ratio with 1 × 10^5^/well OT-I CD8^+^TCRVβ5^+^ T cells, pre-enriched by negative selection with the depletion mAbs (CD19/B220/CD4/Gr-1/CD11b/MHC-II/Ter119). The cells were then treated with 10 μg/ml brefeldin A (BFA), incubated for another 24 hrs and harvested, followed by fixation with 2% paraformaldehyde for 20 min at 4 °C and incubation with fluorochrome-conjugated antibodies, including anti-mouse IL-2-FITC, TNF-α-PE, and IFN-γ-Alexa647, or perforin-FITC and granzyme B-PE, in 0.1% saponin/PBS. The cells were then surface stained with mAbs against CD3ε, CD8, and TCRVα2 (B20.1) or TCRβ5 (MR9-4), followed by cytometric analysis with a FACSVerse flow cytometer (BD Biosciences).

### Fluorescence microscopy

To examine the expression of perforin and granzyme B by fluorescence microscopy, the Gr-1^+^ subsets were plated in complete RPMI 1640 medium containing GM-CSF (5 ng/ml), and treated with PBS or 100 μg/ml PLGA/OVA NPs for 16 hrs, followed by co-culture with the OT-I TCRVα2^+^ CD8^+^ T cells for 48 hrs. The cells were then treated with 10 μg/ml brefeldin A (BFA) for 24 hrs and cytospun at 1,000 rpm for 3 min onto the silane-coated micro glass slides with a Shandon Cytospin4 (Thermo Scientific), followed by fixation with methanol at −20 °C for 5 min and washes with PBS. The non-specific binding was blocked for 5 min with 10% goat serum/PBS. The cells were then incubated at 4 °C for 16 hrs with rat purified anti-mouse perforin (eBioOMAK-D) (1:50) or anti-mouse granzyme B (16G6) (1:100), washed with PBS again, and incubated for 1 hr with goat anti-rat biotin (1:500). After several washes with PBS, the cells were stained for 1 hr with tetramethyl-rhodamineisothiocyanate-conjugated extravidin (extravidin-TRITC) (1:100) and CD8-FITC, washed with PBS, and stained for 5 min with DAPI (1 μg/ml). The cells were washed with PBS again, and the slides were mounted with a fluorescent mounting medium (DakoCytomation) and observed with a Zeiss Axio Imager A1 fluorescence microscope.

### *In vitro* antigen-specific cytotoxicity assays

Cytotoxicity assays were performed using the EL4 target cells, with or without pre-coating with 1 μM SIINFEKL peptide for 1 hr^35^,^36^,^37^. Briefly, the Gr-1^high^ (Lin2¯CD11b^+^Gr-1^high^) and Gr-1^low^ (Lin2¯CD11b^+^Gr-1^low^) subsets were isolated from the bone marrow of naïve mice and sorted by FACS with a BD FACSAria III. The cells were treated with or without the PLGA/OVA NPs for 16 hrs. The cells were then washed with PBS and co-cultured for 72 hrs in complete RPMI 1640 medium containing 10 U/ml IL-2 at 1:1 ratio with the CD8^+^TCRVβ5^+^ T cells isolated and enriched from the spleen of OT-I mice. The effector T cells were then assayed at various effector: target cell (E/T) ratios for cytotoxic activity on the target cells. Briefly, the effectors were co-cultured for 4 hrs at 37 °C at various effector: target cell ratios (E/T = 0.75:1, 1.5:1, 3:1, 6:1, and 12:1) with EL4 cells, with or without being pulsed with 1 μM SIINFEKL in the 96-well plates. The cells were used for cytolytic analysis of lactic dehydrogenase (LDH) with the CytoTox96 non-radioactive cytotoxicity assay kit (Promega) according to the manufacturer’s instruction. After the reaction was stopped, the microplates were centrifuged and fifty μl of the supernatant was collected for the cytotoxicity assay. The amount of LDH release was determined by taking the absorbance at 490 nm, and background absorbance values of phenol-red free culture medium was subtracted from all absorbance values. The percent specific lysis was calculated from triplicate samples as follows:


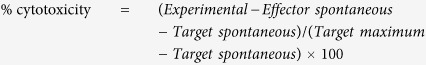


where *Effector spontaneous* and *Target spontaneous* are the spontaneous LDH release by the control OT-I CD8^+^ effector T cells and control target cells (EL4 or SIINFEKL-loaded EL4), respectively. *Target maximum*, on the other hand, was obtained by treatment of EL4 cells, without T cells, for 45 min with 10 μl lysis buffer [9% (v/v) Triton X-100].

### Detection of OVA-specific humoral response by ELISA (enzyme-linked immunosorbent assay)

To detect the subclasses of immunoglobulin (IgG) of the antigen-specific immune response to PLGA NPs, six-to-ten week old C57BL/6J mice were *i.v*. immunized on days 0, 7, 14, 21, and 28 with 100 μl PBS, 100 μl OVA (100 μg/ml), 100 μl PLGA NPs/PBS (1 mg/ml), or 100 μl PLGA-OVA NPs/PBS (1 mg/ml). One hundred microlitres of blood was collected from the submandibular facial veins of mice on days 3, 10, 17, 24, and 31, centrifuged for 10 min at 4,000 rpm, and the supernatant removed for enzyme-linked immunosorbent (ELISA) assay. Briefly, the 96-well ELISA plates were coated overnight with 50 μl OVA (10 μg/ml) in 0.1 M carbonate buffer, pH 9.6. The plates were washed five times with 300 μl washing buffer (0.5% Tween 20 in PBS), blocked for 1 hr with 200 μl blocking buffer containing 0.1% bovine serum albumin (BSA) in PBS, washed again, and then reacted with 100 μl serially diluted sera for 2 hrs. Thereafter, the plates were thoroughly washed and reacted for 1 hr with 1:5000 diluted purified rat anti-mouse IgG1 (RMG1-1), IgG2a (RMG2a-62), or IgG2b (RMG2b-1), washed with PBS, and blocked with goat-anti-rat horseradish peroxidase (HRP), followed by detection with tetramethylbenzidine (TMB) substrate by incubation for 15 min at room temperature. The reaction was stopped with 50 μl 1 M H_3_PO_4_ and read for absorbance at 450 nm with a microplate spectrophotometer (BioTek PowerWave XS, Winooski, VT).

### Adoptive transfer of OT-I CD8^+^ T cells and cell proliferation *in vivo*

To analyse the immune responses induced by the polymeric nanoparticles, CD8^+^ cells from spleens of OT-I mice were enriched by negative selection and labelled with CFSE, as described above. A total of 2 × 10^6^ CFSE-labelled OT-I CD8^+^ T cells were adoptive transferred by tail vein injection into the C57BL/6J recipient mice that were pre-irradiated sub-lethally at a total body irradiation of 600 rad with an IBL-637 (^137^Cs) γ-irradiator (CIS Biointernational, Saclay, France) (day 0). Twenty-four hours later, mice were either left untreated or intravenously injected with 100 μl PBS, 100 μl OVA (100 μg/ml), 100 μl PLGA NPs (1 mg/ml), or 100 μl PLGA/OVA (1 mg/ml) NPs. On day 5 after immunization, cells from the spleen or draining lymph nodes were collected and analysed by flow cytometry.

### Statistical analysis

Data were analyzed by t-test or one-way analysis of variance (ANOVA) using the SigmaPlot software *v*.11.2 (Systat Software, *Inc*.), and presented as mean ± standard error of the mean (SEM).

## Additional Information

**How to cite this article:** Yang, Y.-W. and Luo, W.-H. Recruitment of bone marrow CD11b^+^Gr-1^+^ cells by polymeric nanoparticles for antigen cross-presentation. *Sci. Rep.* **7**, 44691; doi: 10.1038/srep44691 (2017).

**Publisher's note:** Springer Nature remains neutral with regard to jurisdictional claims in published maps and institutional affiliations.
